# Supplementary material for: Covalently linked molecular catalysts in conjugated polymer dots boost photocatalytic alcohol oxidation in neutral condition
Source: Nat Commun. 2024 Aug 8;15:6765. doi: 10.1038/s41467-024-51097-z (PMC11310486; doi:10.1038/s41467-024-51097-z)
Supplement: Supplementary file 1 — Supplementary Information [file 41467_2024_51097_MOESM1_ESM.pdf]

Covalently linked molecular catalysts in conjugated polymer dots boost photocatalytic alcohol oxidation in neutral condition

Sicong Wang[1], Mariia V. Pavliuk[1], Xianshao Zou[2], Ping Huang[1], Bin Cai[1], Orpita M. Svensson[1], Haining Tian[1*]

[1] Department of Chemistry - Ångström Laboratory, Uppsala University, 751 20 Uppsala, Sweden

[2] Qingdao Innovation and Development Base, Harbin Engineering University, Qingdao CN-266 000, China

E-mail: haining.tian@kemi.uu.se

Contents

Characterizations……………………………………………………………..………..3
Materials…………………………………………………………………….…..…..…4
Supplementary methods……………………………………………………..……...4
LED lamp spectrum……………………………………………………………..…...9
Xe lamp spectrum…………………………………………………………..……..9
^{1}H-NMR spectrum of monomer……………………………………………………10
Calibration curves for H_2O_2 and aldehyde detection……………………….…..….11
Spectroscopy studies for excluding physical adsorption of TEMPO…………………...15
Calibration curve for EPR measurement………………………………………………16
DLS results of Pdots…………………………………………………………………….…17
Absorption spectra of polymers in THF……………………………………………...18
Cyclic voltammetry of polymers in THF……………………………………………..19
Fluorescence study of polymers in THF…………………………………………….20
Fluorescence quenching of PFBT-COOH by TEMPO in solutions…………………….21
Absorption spectra of polymers and Pdots with TEMPO in solutions…………….…22
Cyclic voltammetry of polymers on TiO_2 films.……………………………………23
Photoelectrochemical measurements..24
Photocatalysis measurements..25
Scheme of photocatalysis mechansim...26
Stability study of Pdots...27
Singlet oxygen generation...29

Characterizations

The hydrodynamic diameters of samples (Dynamic light scattering (DLS) measurements) were measured by a Zetasizer Nano-S from Malvern Instruments Nordic AB. UV-vis absorption spectroscopy was measured with Shimadzu UV-1900i. Steady-state fluorescence spectroscopy was measured with Edinburgh spectrofluorometer FS5. Cryo-electron microscopy (Cryo-EM) was measured with Zeiss Libra 120 transmission electron microscope. The fluorescence lifetime of samples in THF was measured with Edinburgh spectrofluorometer FS5. The fluorescence lifetime of Pdots was measured with a streak camera (C5680 and M5675, Hamamatsu) with the background and camera sensitivity corrections. The 400 nm excitation laser light was generated by an optical parametric amplifier (OPA) system (Harmony, Fluence) based on a fundamental 1030 nm laser (200 kHz, Jasper 10, Fluence). After excitation, the fluorescence was collected after a long-pass filter and finally ended in a monochromator. The molecular weight of the polymer was determined with an Agilent 1260 Infinity GPC (Gel Permeation Chromatography) system fitted with PolyPore columns and a refractive index detector. PMMA standards were used to calibrate the system. Tetrahydrofuran (THF) was used as the mobile phase with a flow rate of 1 mL min^{-1} and the analysis was performed at 35 °C. The residual Pd concentration was measured by inductively coupled plasma-optical emission spectrometry (ICP-OES), Avio 200, PerkinElmer. The LED spectrum was measured with an Ocean Optics HR2000 Spectrometer. The photocatalytic activity was measured with LED lamp (Zenaro Lighting GmbH, SL-PAR38B/P17/50/E50/ND/27/UNI/EU/ZN, 420 - 750 nm, Spectrum shown in Figure S1). External quantum efficiency (EQE) was measured with a Xe lamp (CEL-HXF300, spectrum shown in Figure S2).

Materials

Ethyl bromoacetate, 2,7-Dibromofluorene (>98 %), 2,7-Dibromo-9,9-di-noctylfluorene(>98 %) and 4,7-Bis(4,4,5,5-tetramethyl-1,3,2-dioxaborolan-2-yl)-2,1,3-benzothiadiazole (>95 %) are obtained from TCI EUROPE N.V. Tetrakis(triphenylphosphine)palladium(0) ($Pd(PPh_3)_4$), N, N-diethyl-p-phenylenediamine (DPD) and peroxidase (POD) are obtained from Sigma-Aldrich. All other chemicals are obtained from Fisher-Scientific (Sweden) and used as received unless indicated otherwise.

Synthesis of PFBT with possibly adsorbed TEMPO

In this process, 50 mg (0.022 mmol) PFBT and 10 mg (0.058 mmol) TEMPO-NH_2 were mixed in 50 mL THF and stirred at room temperature for 24 h. THF was removed by vacuum evaporation and the product was washed with water under sonication for 30 min in 250 mL water for three times to remove the water-soluble TEMPO-NH_2 residuals on the surface. Afterwards, the washed PFBT-COOH was dissolved in THF and precipitated in ethyl easter twice to remove the capsulated TEMPO-NH_2 to get 31 mg product.

Determination of H_2O_2

Photocatalytic generated H_2O_2 was detected by a widely used DPD (N, N-diethyl-p-phenylenediamine) method.[1] Typically, 100 µL reaction solution was collected from reaction cuvette and mixed with 2 mL phosphate buffer solution (pH=5.4). Then, 50 µL of DPD (10 mg mL^{-1}) and 50 µL of POD (1 mg mL^{-1}) solution were added into the mixture successively. The mixture was stored in the dark for 1 min for a complete reaction. The red solution was used to detect the concentration of H_2O_2 by the absorption at 551 nm, calibration plots are shown in Figure S6.

Determination of formaldehyde

The determination of formaldehyde was realized via the Hantzsch reaction between acetylacetone, ammonia and formaldehyde proceeded at pH=6, which is a highly selective reaction towards microgram amounts of formaldehyde.[2] In detail, after the photocatalytic reaction, Pdots were filtered by a filtering centrifuge tube (10KD). Afterwards, 500 µL filtered solution was mixed with 750 µL of acetylacetone and 750 µL of ammonia in 1 mL of 0.5 M PBS at pH=6. The mixture was left at room temperature for 2h and the colour turned from transparent to yellow with an absorption peak appearing at 412 nm. The calibration curve is shown in Figure S7.

Detection of H_2O_2 based on potassium titanium oxalate ($C_4K_2TiO_9$) method

In a typical detection, 200 µL of reaction solution was added to 2 mL of 6 mM $C_4K_2TiO_9$ solution, the solution turns yellow immediately and the absorption at 382 nm was used to determine the concentration of H_2O_2 (calibration curve shown in Figure S8). This method is used for H_2O_2 detection in photocatalysis systems containing benzoquinone due to its selectivity.

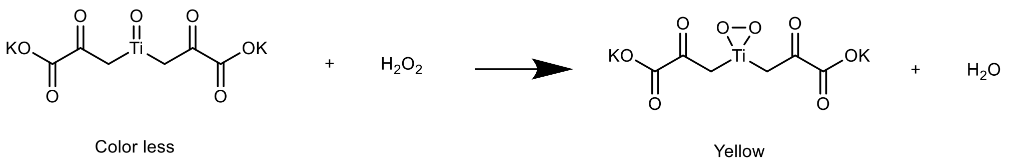

Supplementary Fig. 1. Detection mechanism of H_2O_2. $C_4K_2TiO_9$ method for detection of H_2O_2.

Photocatalytic benzyl alcohol oxidation

2 mL Pdots reaction solution (22 µg mL^{-1}) which contains 0.19 M benzyl alcohol was purged by O_2 for 15 min to ensure the saturation of O_2 in solution. Afterwards, the cuvette was irradiated by a LED lamp (Zenaro Lighting GmbH, SL-

PAR38B/P17/50/E50/ND/27/UNI/EU/ZN, 420 - 750 nm) with an irradiation density of 50 mW cm^{-2}. The irradiation density was measured with a PM100D power meter (Thorlabs). After 2 h irradiation, Pdots were removed by centrifuging with Amicon® Ultra Centrifugal Filter (MWCO 1k Da). The solution was diluted 20 times to lower the concentration of benzyl alcohol. The diluted solution was then detected by LC-MS and the peak eluting at 4.2 min and was used to determine the concentration of benzaldehyde.

Photocatalytic ethanol oxidation

2.5 mL Pdots reaction solution (22 µg mL^{-1}) which contains 5 M ethanol was purged by O_2 for 15 min to ensure the saturation of O_2 in solution. Afterwards, the cuvette was irradiated by a LED lamp (Zenaro Lighting GmbH, SL-PAR38B/P17/50/E50/ND/27/UNI/EU/ZN, 420 - 750 nm) with an irradiation density of 50 mW cm^{-2}. The irradiation density was measured with a PM100D power meter (Thorlabs). After 2 h irradiation, the concentration of acetaldehyde was determined by the Purpald method (shown below). Briefly, 200 uL of the reaction solution was added to 2 mL of 10 mM 4-Amino-3-hydrazino-5-mercapto-1,2,4-triazole (Purpald) solution (containing 0.3 M NaOH), the solution colour changes from light yellow to purple after 30 min and the absorption peak at 536 nm was used to determine the concentration of acetaldehyde (Figure S9).

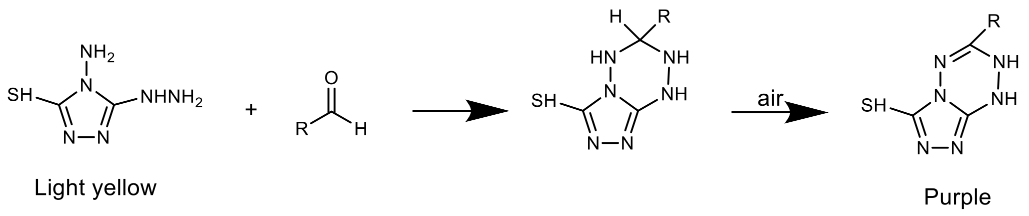

Supplementary Fig. 2. Detection mechanism of aldehydes. Purpald method for detection of aldehydes.

Quantum Yield Measurement

External quantum efficiency (EQE) of PFBT-T-PCBM Pdots was measured under the same condition as for photocatalytic H_2O_2 generation except that the concentration of Pdots was 90 µg mL^{-1}. The light source was replaced by Xenon lamp (CEL-HXF300) with a light filter (QD 450 nm) and the irradiation density was 2.7 mW cm^{-2}. The irradiation density was measured with a PM100D power meter (Thorlabs). EQE was calculated by the equation below:

$$\mathrm{EQE} = \frac{2 * generated\ H_2O_2\ molecules}{incident\ photons\ (N)} \quad (1)$$

$$\mathrm{N} = \frac{It\lambda}{hc} \quad (2)$$

Where N represents the incident photons, *I* represents the irradiation density, *t* is the irradiation time, *λ* is the wavelength of the incident light, *h* is the plank constant and *c* is the speed of light.

Estimation of band gaps and energy level calculation of polymers

The optical bandgap E_g of polymers, was estimated by zero-zero transition energy (E_{0-0}), which is calculated by eqn (3).[3] Where h is Plank's constant, c is the speed of light and λ is the wavelength.

$$E_{0-0} = \frac{hc}{\lambda} \quad (3)$$

λ, ca. 497 nm for PBFT-T, is determined as the intersection point of the UV-vis spectra and the steady-state emission spectra of PFBT-T in THF. Therefore, the optical band gap of PFBT-T is calculated to be 2.49 eV.

The reduction potential ($E_{PFBT-T/PFBT-T^-}$) of PFBT-T was measured to be -0.95 V vs. NHE in THF solution with CV measurements (Figure S14).[3] Reduction potential of the excited PFBT-T ($E_{PFBT-T^*/PFBT-T^-}$) was calculated based on equation below[3]:

$$E_{PFBT-T^*/PFBT-T^-} = E_{PFBT-T/PFBT-T^-} + E_{0-0} \quad (4)$$

Therefore, ($E_{PFBT-T^*/PFBT-T^-}$) was calculated to be 1.54 V vs. NHE.

Calculation of charge transfer efficiency and rate

Taking PFBT-T/PCBM as an example, according to equ. (5) and (6), the charge transfer efficiency (η_{CT}) and charge transfer rate (τ_{CT}^{-1}) can be calculated respectively.

$$\eta_{CT} = 1 - \frac{\tau_{PFBT\text{-}T/PCBM}}{\tau_{PFBT\text{-}T}} \quad (5)$$

$$\frac{1}{\tau_{PFBT\text{-}T/PCBM}} = \frac{1}{\tau_{CT}} + \frac{1}{\tau_{PFBT\text{-}T}} \quad (6)$$

τ_{CT} and $\tau_{PFBT\text{-}T/PCBM}$ are the fluorescence lifetime of PFBT-T and PFBT-T/PCBM which are calculated by fitting the time-resolved fluorescence spectra.

Supplementary Table 1. Lifetime parameters of PFBT-COOH, PFBT-T, PFBT-COOH/PCBM and PFBT-T/PCBM Pdots.

Sample	τ_1 (ps)	Rel_1 (%)	τ_2 (ps)	Rel_2 (%)	τ_{ave} (ps)
PFBT-COOH	80	39	415	61	285
PFBT-T	73	44	370	56	240
PFBT-COOH/PCBM	31	81	295	19	81
PFBT-T/PCBM	23	90	198	10	41

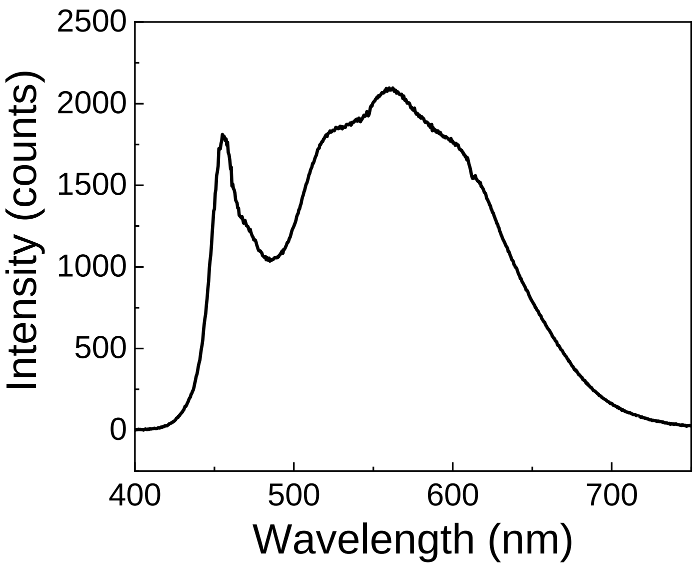

Supplementary Fig. 3. LED lamp spectrum. Spectrum of LED lamp used for photocatalysis.

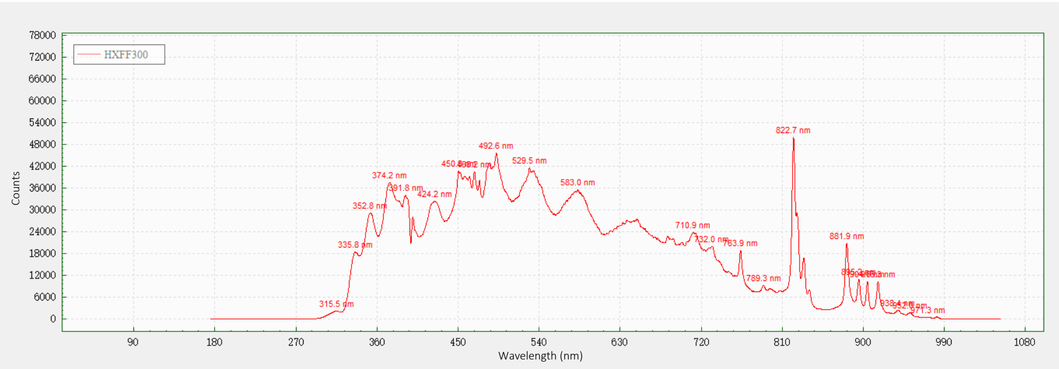

Supplementary Fig. 4. Xe lamp Spectrum. Spectrum of Xe lamp that used for external quantum yield measurement. (provided by *Beijing China Education Au-light Co., Ltd*.)

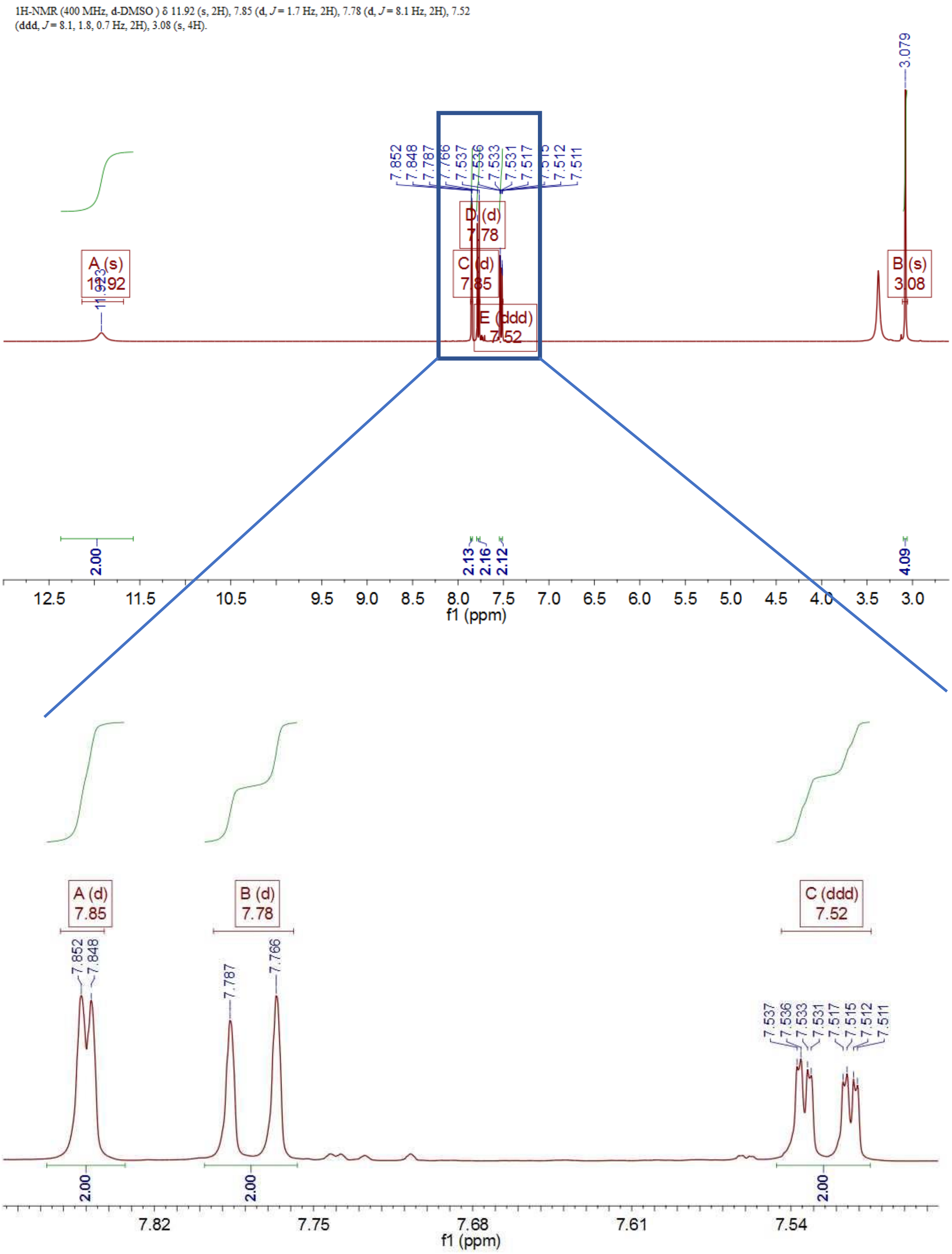

Supplementary Fig. 5. ^{1}H-NMR spectrum. Synthesized monomer 2,2'-(2,7-dibromo-9H-fluorene-9,9-diyl)diacetic acid.

The peaks at 7.52, 7.78 and 7.85 ppm are attributed to H atoms on benzene rings and the peak at 11.92 ppm is attributed to the H atom in carboxyl groups. The ratio of benzene ring H atoms to carboxyl group H atoms is around 3:1, indicating that two carboxyl groups are linked to each 2,7-dibromofluorene unit.

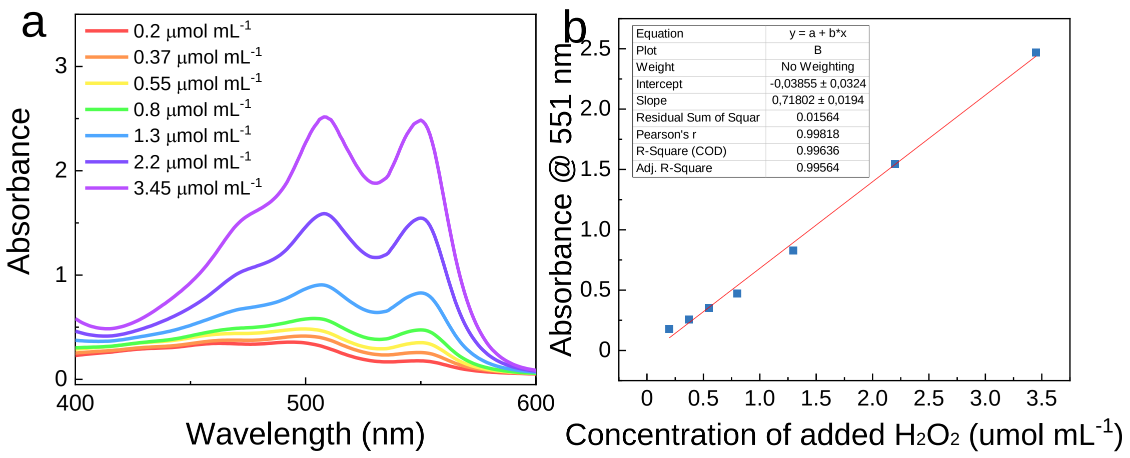

Supplementary Fig. 6. Calibration curves. H_2O_2 detection by DPD and POD method, (a) absorption spectra with various H_2O_2 concentration. (b) fitted results with absorbance at 551 nm.

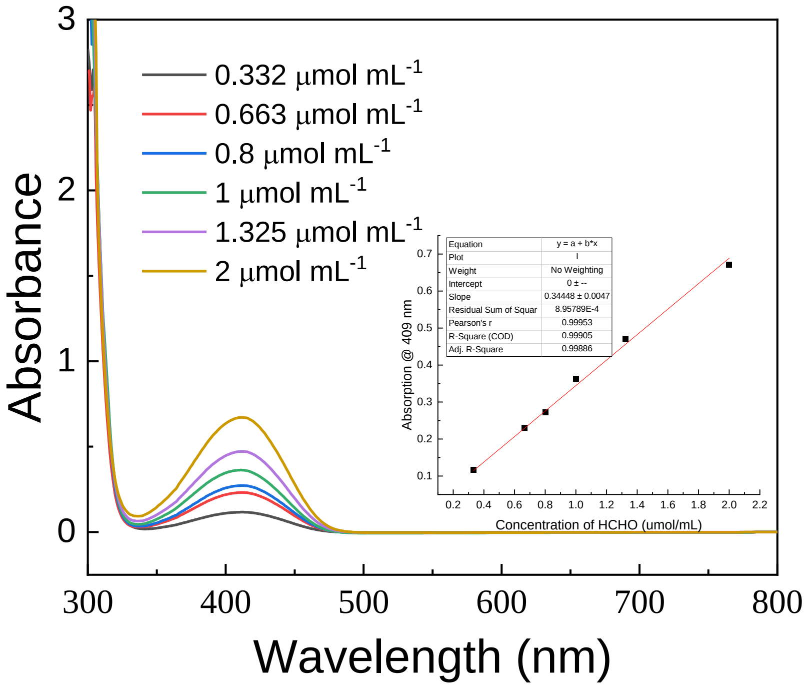

Supplementary Fig. 7. Calibration curve. Detection of formaldehyde.

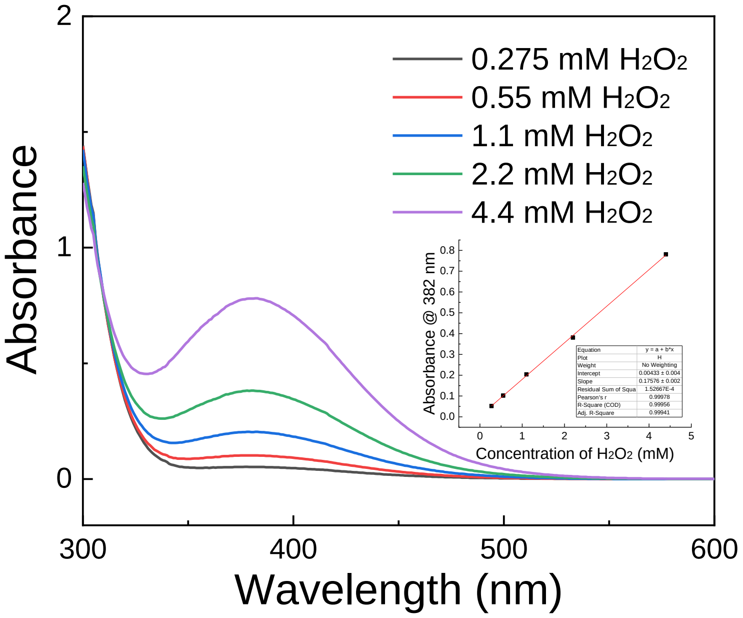

194 **Supplementary Fig. 8. Calibration curve.** Detection of H_2O_2 by $C_4K_2TiO_9$ method.

195

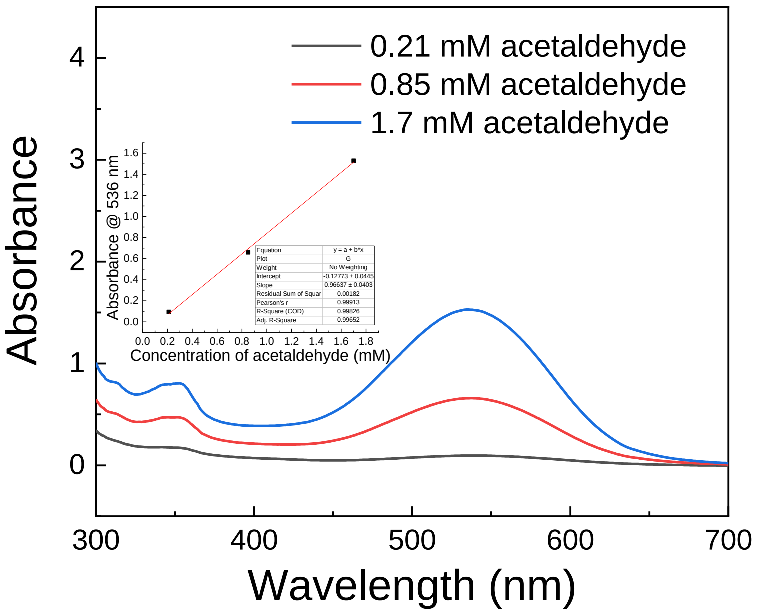

Supplementary Fig. 9. Calibration curve. Detection of acetaldehyde by purpald method.

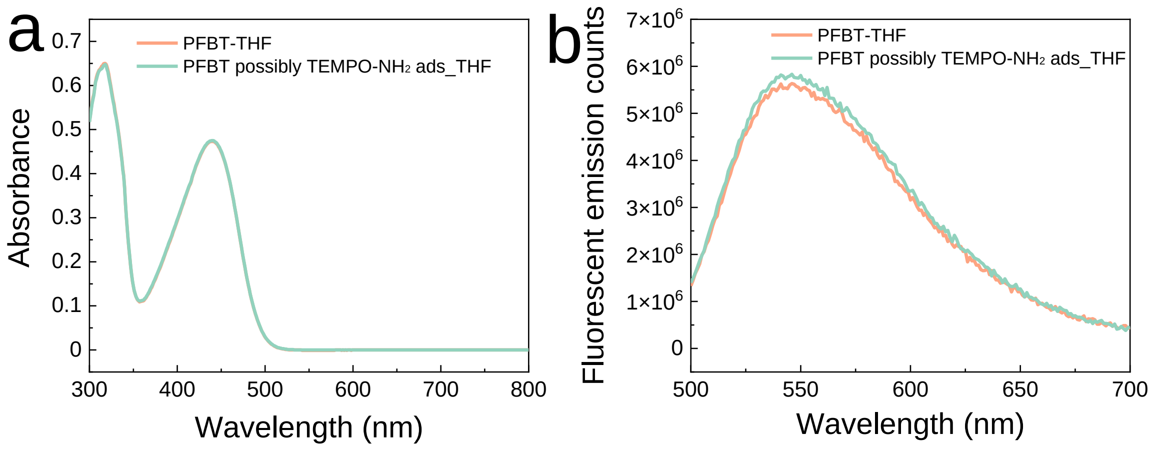

Supplementary Fig. 10. Steady-state absorption and fluorescence emission. Absorption (a) and fluorescent emission (b) of PFBT with possibly adsorbed TEMPO-NH_2.

As shown in Figure S8a, no shift in absorption has been observed after synthesis, excluding the possibility of covalent grafting of TEMPO in this situation. According to the steady-state quenching results in Figure S8b, the adsorption of TEMPO-NH_2 molecules on PFBT is excluded since no quenching on fluorescence intensity was observed.

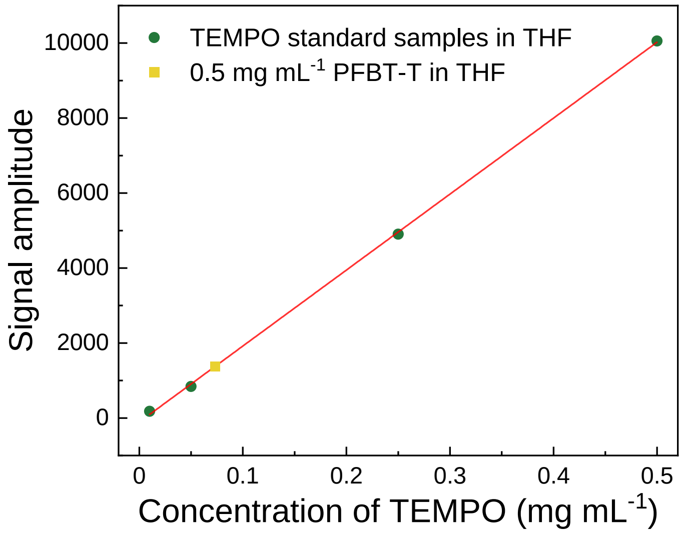

Supplementary Fig. 11. Calibration curve. EPR signal of various TEMPO radical concentration in THF solution and the measured result of 0.5 mg mL^{-1} PFBT-T in THF.

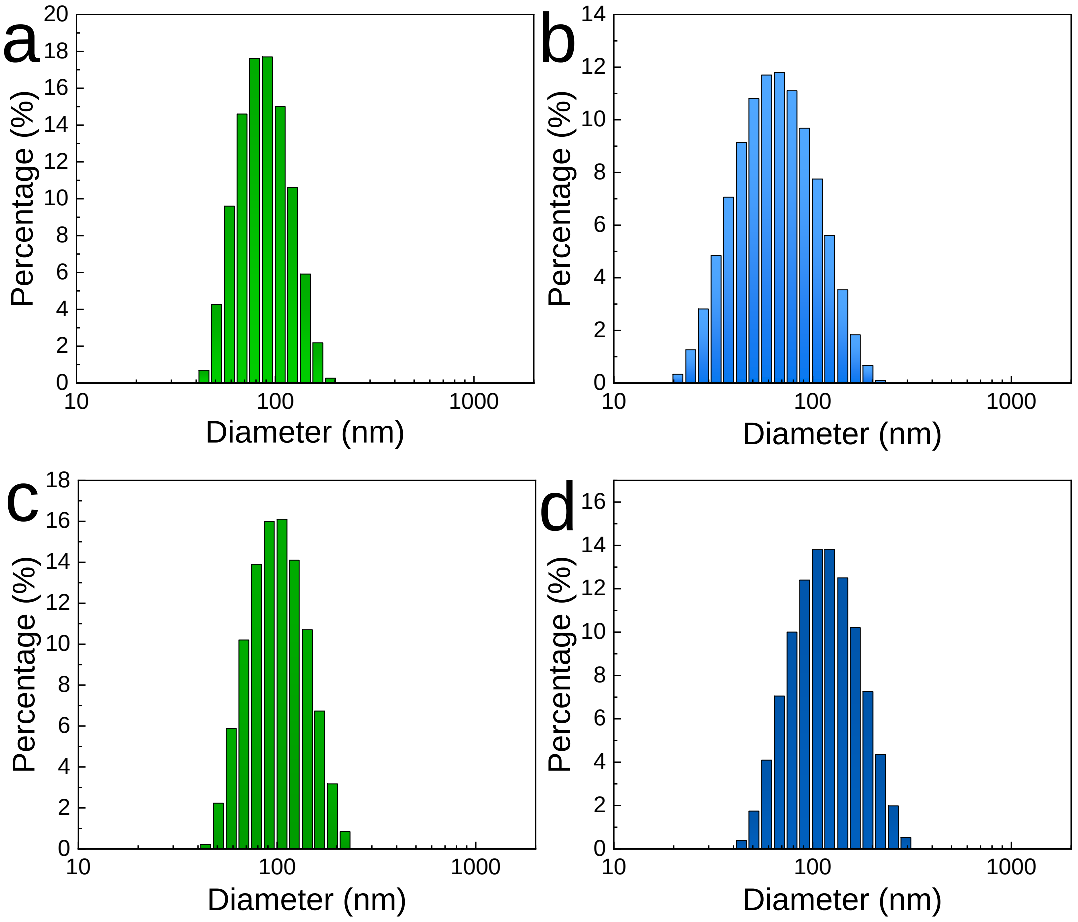

Supplementary Fig. 12. DLS spectra. (a) PFBT-COOH Pdots, (b) PFBT-T Pdots, (c) PFBT-COOH-PCBM Pdots and (d) PFBT-T-PCBM Pdots.

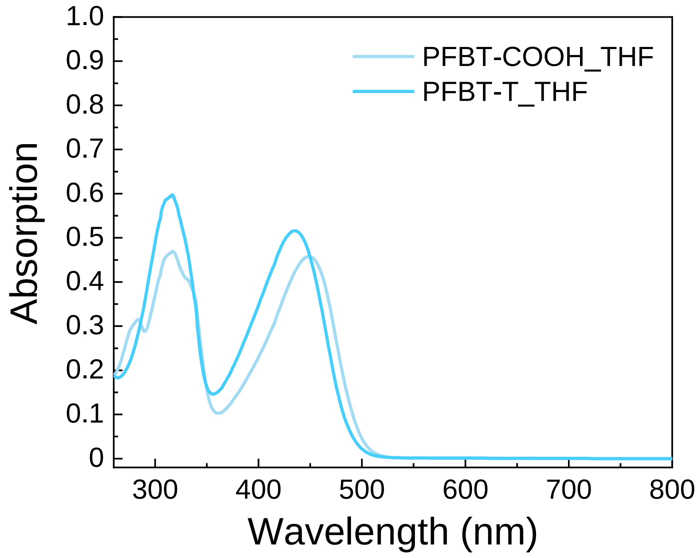

Supplementary Fig. 13. Absorption spectra. PFBT-COOH and PFBT-T in THF solution.

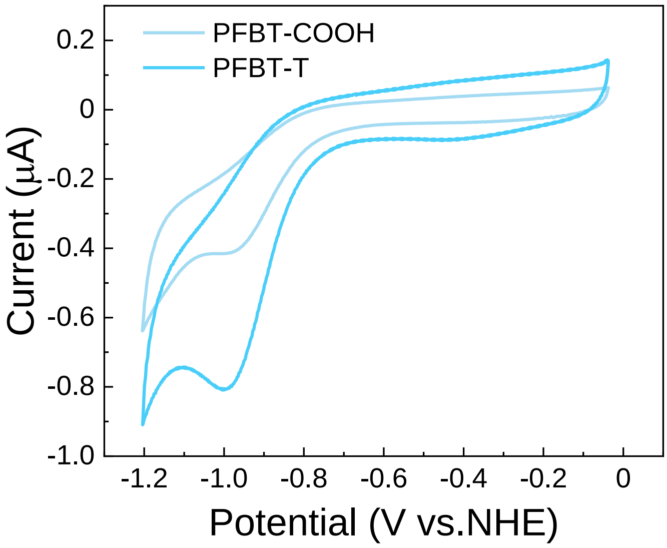

Supplementary Fig. 14. Cyclic voltammetry. PFBT-COOH and PFBT-T in THF with 0.1 M $TBAPF_6$, scan rate 50 mV s^{-1}.

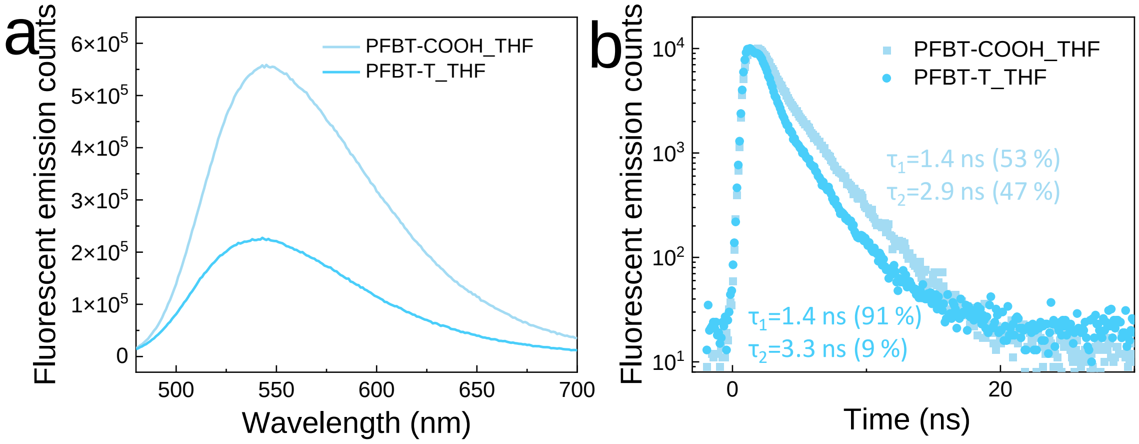

Supplementary Fig. 15. Steady-state fluorescence and time-resolved fluorescence. (a) Steady-state fluorescence of PFBT-COOH and PFBT-T in THF solution, excited at 420 nm. (b) Time-resolved fluorescence emission spectra and fitted lifetime of PFBT-COOH and PFBT-T in THF solution, excited by 420 nm laser.

The quenching efficiency calculated from fluorescence lifetime is much lower than that of steady-state quenching efficiency in both THF and Pdots. This can be explained by a non-fluorescent compound formed between grafted TEMPO molecules and PFBT polymer backbones, resulting in a lower fluorescence intensity but not contributing to a shorter fluorescence lifetime.

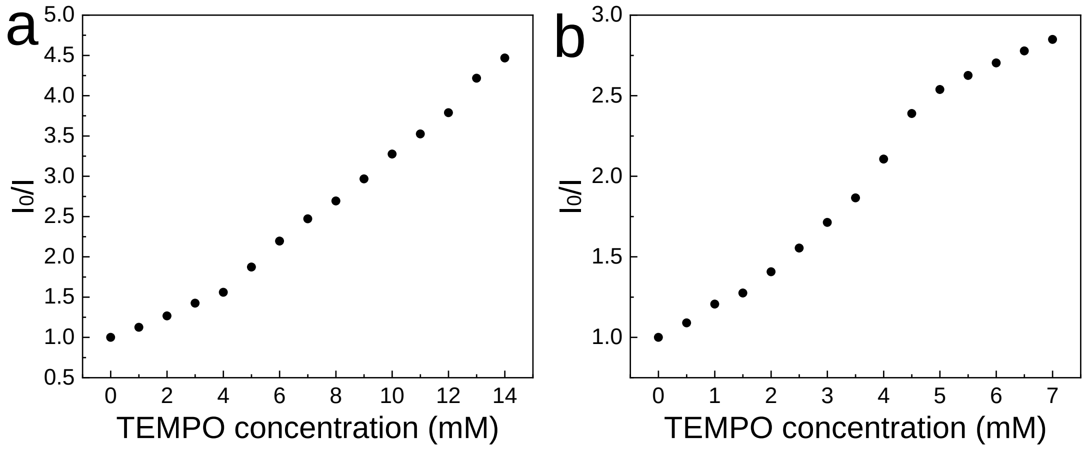

Supplementary Fig. 16. Stern-Volmer plots. Quenching of fluorescence of PFBT-COOH in (a) THF and (b) Pdots by TEMPO solution.

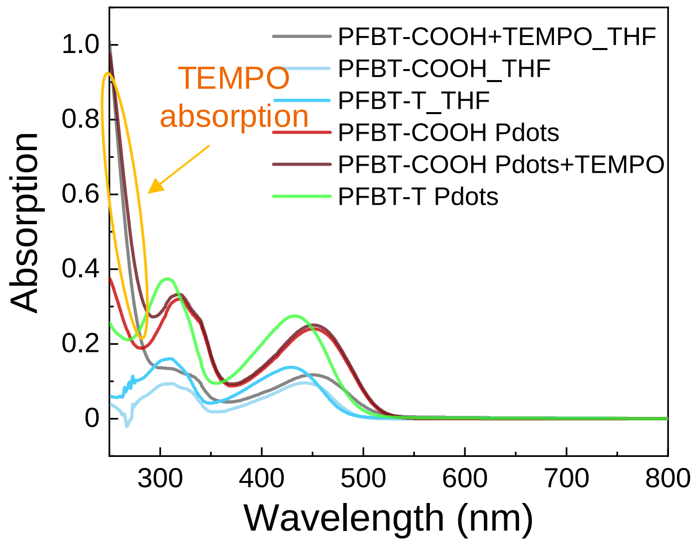

248
249 **Supplementary Fig. 17. Absorption spectra.** PFBT-COOH, PFBT-T, added free
250 TEMPO in THF, PFBT-COOH Pdots, PFBT-T Pdots and added free TEMPO in Pdots
251 solutions.
252

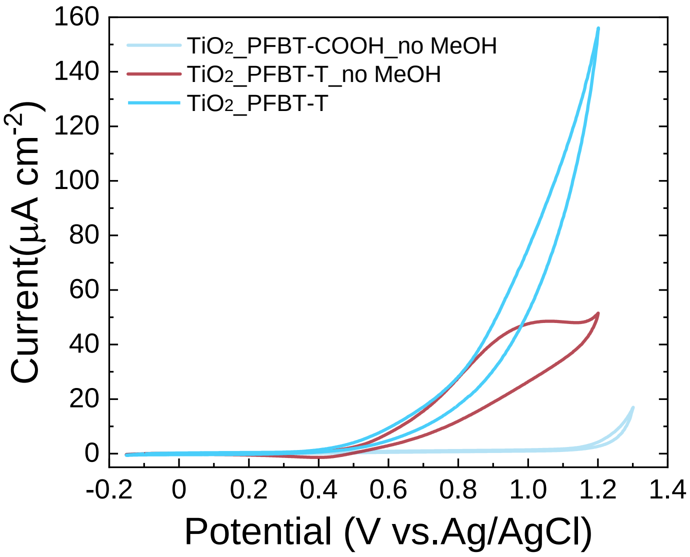

Supplementary Fig. 18. Cyclic voltammetry. PFBT-COOH and PFBT-T coated TiO_2 film on FTO glass in 0.5 M phosphate buffer at pH=6.9, scan rate is 25 mV s^{-1}.

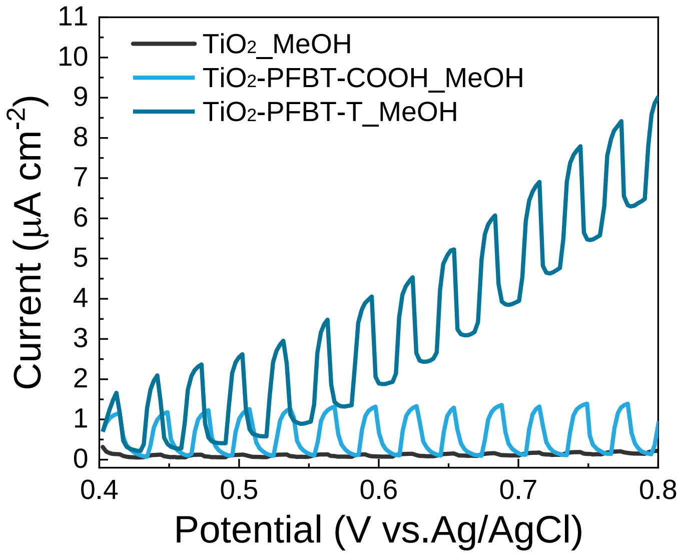

Supplementary Fig. 19. Photoelectrochemical measurements. PFBT-COOH and PFBT-T spincoated on mesoporous TiO_2 film in 0.5 M phosphate buffer (pH=6.9) 5 M MeOH solution, the scan rate was 2 mV s^{-1}, the light was chopped every 5 s.

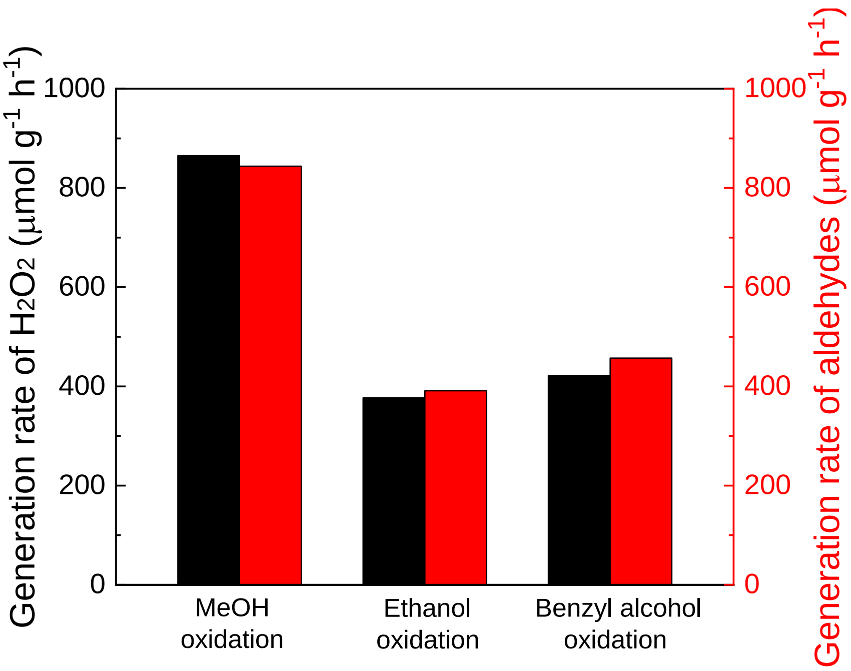

Supplementary Fig. 20. Photocatalysis measurements. PFBT-T/PCBM Pdots used in photocatalytic oxidation of various alcohols.

MeOH, ethanol and benzyl alcohol were used in PFBT-T/PCBM Pdots system to evaluate the universality of the proposed designing strategy. Despite the concentration of benzyl alcohol (0.19 M) being ~ 25 times lower than that of ethanol (5 M), the photocatalytic oxidation rate of benzyl alcohol is higher than that of ethanol. The redox potential for oxidation of benzyl alcohol to benzaldehyde (-0.14 V Vs. NHE)[4] is lower than that for ethanol to acetaldehyde (0.084 V vs. NHE).[5] Moreover, the large overpotential caused by the slow kinetics of coupled electron and proton transfer from C-H and O-H moieties, which requires a potential greater than 1.60 V vs. NHE, is proved to be overcome by TEMPO catalysts.[6] Therefore, the strategy of TEMPO grafting is practical in various alcohol oxidations.

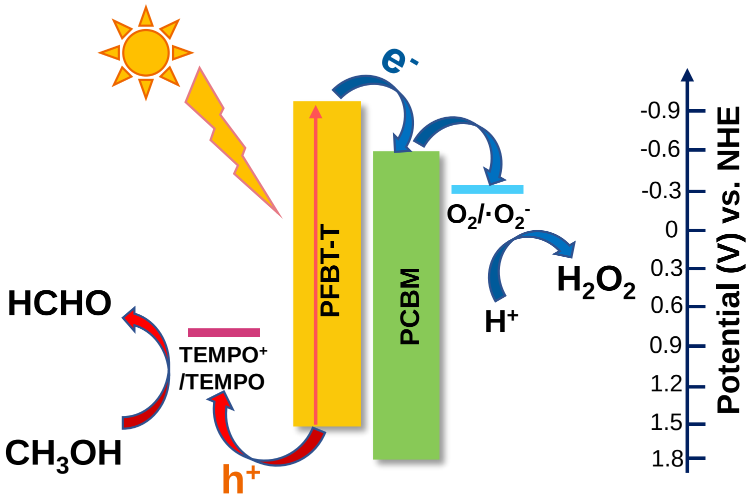

Supplementary Fig. 21. Reaction mechanism. Photocatalytic H_2O_2 and formaldehyde production mecahnism withPFBT-T/PCBM Pdots in neutral conditions.

Supplementary Table 2. Results of superoxide scavenger experiments

Benzoquinone (BQ)	O_2	H_2O_2 generation rate (μmol g^{-1} h^{-1})	HCHO generation rate (μmol g^{-1} h^{-1})
None	Saturated	865	844
10 mM	Saturated	0	797
10 mM	None	0	113

By adding benzoquinone (BQ) as the scavenger of superoxide radicals ($O_2^{\cdot-}$), the H_2O_2 generation with PFBT-T/PCBM Pdots was completely inhibited while the formaldehyde production rate remained. In order to rule out the direct electron transfer from Pdots to BQ completely competes the formation of superoxide, an additional control experiment has been done in presence of BQ, but in the absence of oxygen. In this case, the result shows that HCHO is formed, but with much lower generation rate, only ca. 13% of the rates from the systems with O_2. This proves that the inhibited generation of H_2O_2 in presence of BQ and O_2 was dominantly from the consumption of the formed superoxide radicals by BQ, meaning that the photocatalytic H_2O_2 should go through a two-step one-electron transfer pathway with $O_2^{\cdot-}$ as an intermediate.

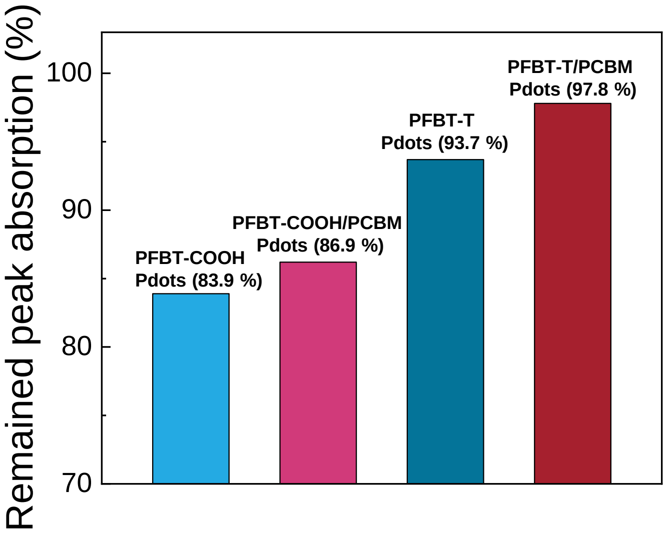

Supplementary Fig. 22. Stability study. Degradation of PFBT-COOH, PFBT-COOH/PCBM, PFBT-T and PFBT-T/PCBM Pdots Pdots systems during 2 h photocatalysis.

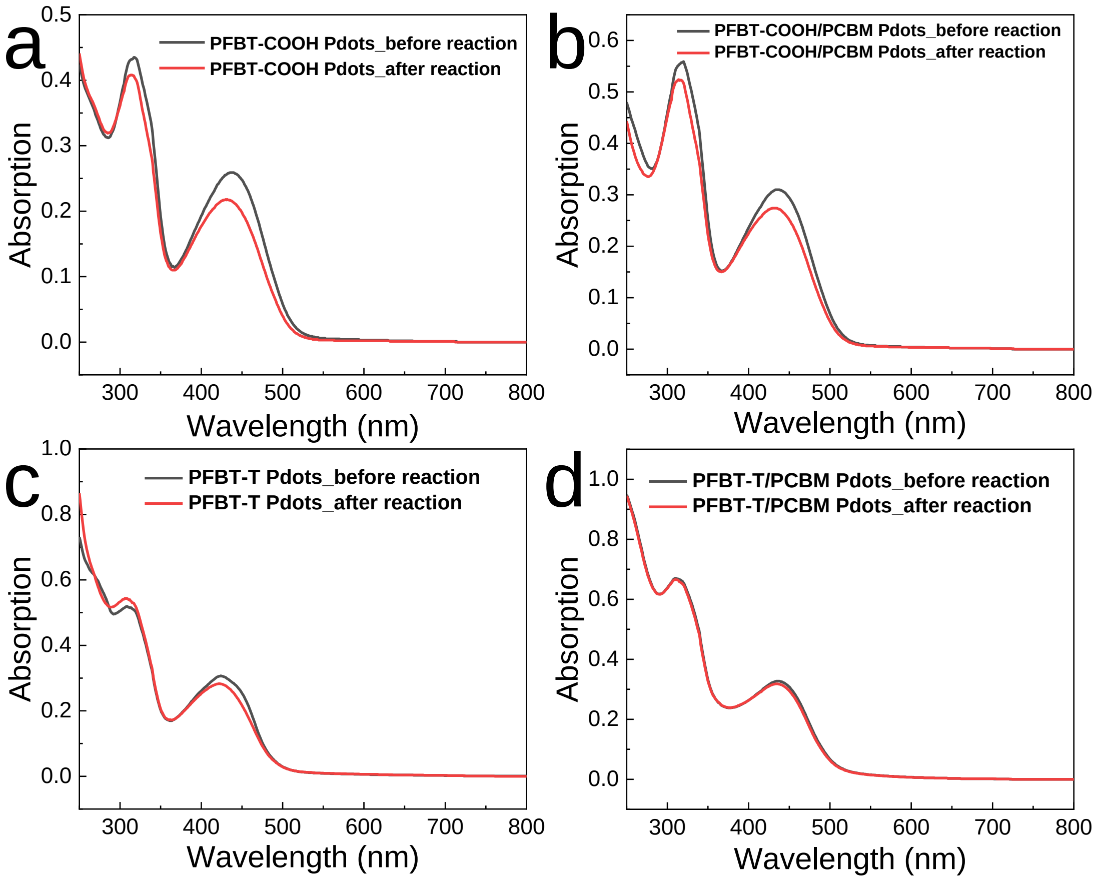

Supplementary Fig. 23. Stability study. Absorption of (a) PFBT-COOH, (b) PFBT-COOH/PCBM, (c) PFBT-T and (d) PFBT-T/PCBM Pdots before and after 2 h reaction.

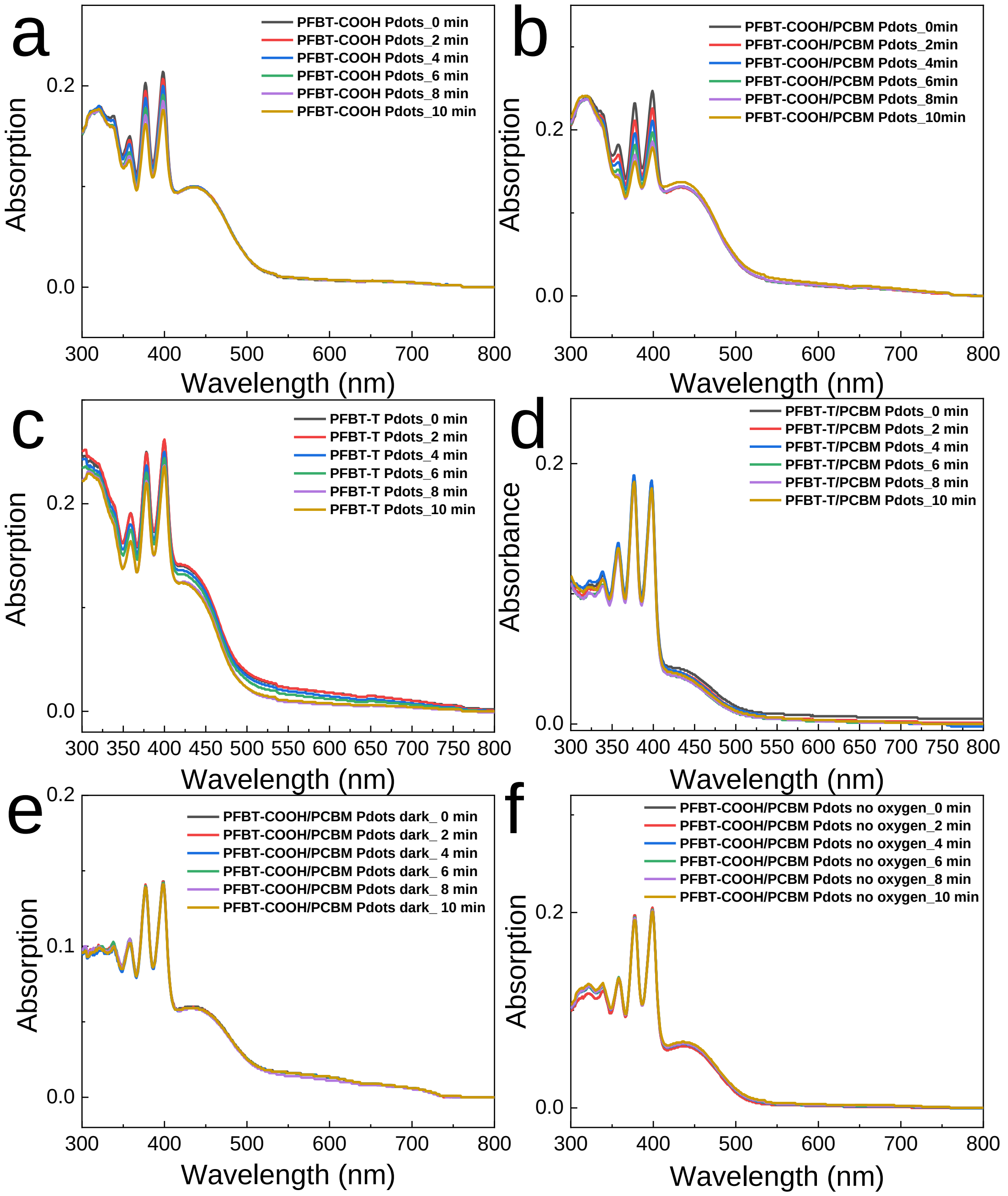

Supplementary Fig. 24. Singlet oxygen generation study. ABDA degradation using (a) PFBT-COOH Pdots, (b) PFBT-COOH/PCBM Pdots, (c) PFBT-T Pdots and (d) PFBT-T/PCBM Pdots under the photocatalysis condition, (e) PFBT-COOH/PCBM Pdots under the dark and (f) PFBT-COOH/PCBM Pdots without oxygen.

As discussed in our previous work,[7] the formation of singlet oxygen is competing with oxidation of MeOH because consumption of the generated holes in Pdots by MeOH will suppress the Dexter energy transfer pathway to form singlet oxygen.[8] In PFBT-T and PFBT-T/PCBM systems, the generation rate of singlet oxygen

is slower than PFBT and PFBT/PCBM systems, which is contributed by faster MeOH oxidation from grafted TEMPO in PFBT-T and PFBT-T/PCBM.

Supplementary References

(1) Wei, Y.; Zhang, J.; Zheng, Q.; Miao, J.; Alvarez, P.; Long, M., Quantification of photocatalytically-generated hydrogen peroxide in the presence of organic electron donors: Interference and reliability considerations. *Chemosphere* **2021**, 279, 130556.

(2) Doroodmand, M.; Kharekani, M., Selective removal of formaldehyde based on Hantzsch reaction using acetylacetone/ammonia-modified mixed matrix absorbent as a novel absorber. *Chem. Eng. J.* **2016**, 283, 453-461.

(3) Pavliuk, M.; Wrede, S.; Liu, A.; Brnovic, A.; Wang, S.; Axelsson, M.; Tian, H., Preparation, characterization, evaluation and mechanistic study of organic polymer nano-photocatalysts for solar fuel production. *Chem. Soc. Rev.* **2022**, 51, 6909.

(4) Hosseini, S.; Janusz, J.; Tanwar, M.; Pendergast, A.; Neurock, M.; White, H., Oxidation by Reduction: Efficient and Selective Oxidation of Alcohols by the Electrocatalytic Reduction of Peroxydisulfate. *J. Am. Chem. Soc.* **2022**, 144, 21103-21115.

(5) Al-Azri, Z.; Chen, W.; Chan, A.; Jovic, V.; Ina, T.; Idriss, H., The roles of metal co-catalysts and reaction media in photocatalytic hydrogen production: Performance evaluation of M/TiO_2 photocatalysts (M = Pd, Pt, Au) in different alcohol–water mixtures. *J. Catal.* **2015**, 329, 355-367.

(6) Nutting, J.; Rafiee, M.; Stahl, S., Tetramethylpiperidine N-Oxyl (TEMPO), Phthalimide N-Oxyl (PINO), and Related N-Oxyl Species: Electrochemical Properties and Their Use in Electrocatalytic Reactions. *Chem. Rev.* **2018**, 118, 9, 4834-4885.

(7) Wang, S.; Cai, B.; Tian, H., Efficient Generation of Hydrogen Peroxide and Formate by an Organic Polymer Dots Photocatalyst in Alkaline Conditions. *Angewandte Chemie International Edition* **2022,** *61* (23), e202202733.

(8) Zhou, N.; Zhu, H.; Li, S.; Yang, J.; Zhao, T.; Li, Y.; Xu, Q., Au Nanorod/ZnO Core–Shell Nanoparticles as Nano-Photosensitizers for Near-Infrared Light-Induced Singlet Oxygen Generation. *J. Phys. Chem. C* **2018**, 122 (14), 7824–7830.
